# Impact of the COVID‐19 virus outbreak on 24‐h movement behaviours among children in Saudi Arabia: A cross‐sectional survey

**DOI:** 10.1111/cch.12999

**Published:** 2022-03-23

**Authors:** Yazeed A. Alanazi, Anne‐Maree Parrish, Anthony D. Okely

**Affiliations:** ^1^ Early Start and School of Health & Society University of Wollongong Wollongong NSW Australia; ^2^ College of Sport Sciences and Physical Activity King Saud University Riyadh Saudi Arabia; ^3^ Illawarra Health and Medical Research Institute University of Wollongong Wollongong NSW Australia

**Keywords:** child, COVID‐19, physical activity, sedentary screen time, sleep

## Abstract

**Background:**

In March 2020, the World Health Organization (WHO) declared the coronavirus (COVID‐19) outbreak as a pandemic. This led many governments to place restrictions on population movement to aid in pandemic control. These restrictions were expected to produce some type of impact on the daily lives of children and their families. The purpose of this study was to investigate the impact of COVID‐19 on 24‐h movement behaviours among Saudi children aged 6–12 years, during the pandemic.

**Methods:**

An online survey of Saudi parents (*n* = 1021) was conducted between 1 October to 11 November 2020 to gather information about the impact of the COVID‐19 outbreak on children's 24‐h movement behaviours, parent and child factors that may be associated with movement behaviours, and perceived changes in children's movement behaviours.

**Results:**

Only 3.4% of Saudi children met all components of 24‐h movement guidelines. Compared with before COVID‐19, children's PA levels declined, they slept more, and their use of electronic screen devices significantly increased. The perceived changes in PA and SB were more unfavourable among girls than boys. Children of older parents, mothers, and those with lower education levels and lower monthly incomes were more likely to meet 24‐h movement guidelines.

**Conclusion:**

The COVID‐19 virus outbreak unfavourably affected Saudi children's movement behaviours, more specifically, girls, which should be taken into account in future research. The results provide an insight into what has changed because of the COVID‐19 restrictions and could be considered as part of the response strategies in Saudi Arabia.

**Key Messages**

- This research examined the impact of COVID‐19 on 24‐h movement behaviours among Saudi children during the COVID‐19 pandemic.
- Children's PA levels declined, they slept more, and their use of electronic screen devices significantly increased.
- The perceived changes in PA and SB were more unfavourable among girls than boys.
- The Ministry of Education, parents, and children need to work together to address the adverse impact of COVID‐19 on Saudi children's movement behaviours.

## 1 INTRODUCTION

In March 2020, the World Health Organization (WHO) declared the coronavirus (COVID‐19) outbreak as a pandemic (WHO, 2020). Many governments placed restrictions on population movement to aid in pandemic control (Han et al., 2020). These restrictions likely impacted children's physical activity (PA), sedentary behaviour (SB), and sleep. Children spent less time outdoors, which is associated with PA levels (Mitra et al., 2020). School closures, which affected more than 1.5 billion children globally (Bates et al., 2020), also reduced opportunities for PA (Guan et al., 2020).

COVID‐19 restrictions affected children's opportunities to meet 24‐h movement guidelines (Guerrero et al., 2020). Studies have found that during COVID‐19, most children did not meet PA or screen time (ST) guidelines, and there was an increase in ST, social media use and in sleep duration, compared with before COVID‐19 (Al Hourani et al., 2021; Kovacs et al., 2021; Moore et al., 2020; Xiang et al., 2020).

More than 140 million children live in the 22 Arabic countries, including Saudi Arabia (Child Labour in Arab States & International Programme on the Elimination of Child Labour (IPEC), n.d.). Across these countries, childhood obesity and sedentary behaviour levels are high and increasing (Farrag et al., 2017; Sharara et al., 2018). Further, there is a lack of evidence from these countries on the impact of COVID‐19 on 24‐h movement behaviours and on important health outcomes (obesity, executive function, motor development, and bone health) across the years of primary school. Assessing the impact of COVID‐19 during the elementary school years is important to monitor prevalence and changes in adequate motor development (Simons et al., 2008; Valentini, 2012).

To our knowledge, no studies have examined the impact of COVID‐19 on movement behaviours among school‐aged children in an Arabian country. By January 2022, the number of COVID‐19 cases has exceeded 575,293 in Saudi Arabia with more than 8892 deaths (Johns Hopkins University, 2021). These are the highest of any Arabian country and have prompted long‐term government restrictions such as school closures and home quarantines. Schooling was conducted remotely for a long period of time (from March to November 2020), and this, along with the other restrictions imposed, likely resulted in changes in the time spent in movement behaviours. Given the high and increasing rates of child obesity in Saudi Arabia, these changes due to COVID‐19 could have significant impact on children's health and development. Therefore, the purpose of this study was to investigate the impact of the COVID‐19 outbreak on 24‐h movement behaviours among Saudi children aged 6–12 years.

## 2 METHODS

This online cross‐sectional survey aimed to recruit children from all 13 regions of Saudi Arabia. The survey was promoted to parents through the Saudi Ministry of Education and on social media.

### 2.1 Data collection

Eligible parents who resided in Saudi Arabia with a child aged between 6 and 12 years completed the survey between 1 October to 11 November 2020. The survey comprised (a) parental and child demographics, (b) child's movement behaviours, and (c) changes in the child's movement behaviours due to COVID‐19. Questions used in the current analyses are shown in Table 1. An online parent information sheet and consent form were completed before commencing the survey. Parents with more than one child were asked to complete a separate questionnaire for each child.

**TABLE 1:** Selected items used in the current analysis from the child survey during COVID‐19 virus outbreak

Survey details	
**Module of child and caregiver background** Option response	
What is the date of birth of the child? What is the (parent/caregivers) date of birth? What is the sex of the child? Parent/caregivers relationship to the child participating in the study? In which region in Saudi Arabia do you live? What is the highest level of parental education? What is your monthly income (SAR)?	(dd/mm/yyyy) [dropdown, boy/girl] [dropdown, specify]
**Current movement behaviours**	
Over the past 7 days, on how many days was your child physically active for a total of at least 60 min per day? During the past 7 days, on how many days did your child do activities to strengthen their muscles and bones? Over the past 7 days, on how many days did your child watch TV/videos/internet using a smart phone or tablet or play video or computer games for entertainment for less than 2 h while sitting or lying down? During the past 7 days, on average how much time per day did your child play outside? How many hours of sleep does your child get in a typical 24‐h/day (including naps)? On a scale of 1 to 7, with the higher number indicating higher quality, how would you rate the quality of your child's sleep?	[dropdown, 0–7 days] [dropdown, 0–7 h] [dropdown, h/min] [dropdown, 0–7/Don't know]
**Change in movement behaviours** Compared with before the COVID‐19 outbreak and related restrictions:	
My child is doing physical activities or sport outside? My child is doing physical activities or sport inside? My child watches TV, movies, uses the computer for leisure or plays sedentary video games? My child uses social media? My child sleeps?	‐ A lot less ‐ A little less ‐ About the same ‐ A little more ‐ A lot more
My child's sleep quality is? The balance of my child's overall healthy movement behaviours (i.e., physical activity, sedentary behaviours, and sleep) are?	‐ A lot worse ‐ A little worse ‐ About the same ‐ A little better ‐ A lot better
As a result of the COVID‐19 outbreak and related restrictions: Is there an inside leisure activity or hobby that your child is doing a lot more now? Is there an outside leisure activity or hobby that your child is doing a lot more now? Has there been a decrease in your child's health (e.g., existing condition worsened or new condition developed)?	‐ Yes [specify] ‐ No

*Note*: Full survey is available in the Supporting Information.

### 2.2 Survey

The survey was based on a parental survey of young children's movement behaviours during COVID‐19 (Okely et al., 2021) and the Children and Youth Movement and Play Behaviours Survey (Moore et al., 2020). It was translated into Arabic and back‐translated into English to ensure appropriateness of the questions. Approval was obtained from the Ministry of Education in Saudi Arabia (42640/2020) and the Human Research Ethics Committee at The University of Wollongong (HE288/2021).

Child and parent birth, sex, region of residence, parental education, and income were assessed using standard questions. PA duration was assessed through parents' responses to questions related to their children's time spent playing outside, doing activities to strengthen their muscles and bones, and being physically active for a total of ≥60 min per day in the past 7 days. SB was assessed through questions related to child's time spent watching TV, using a smart phone, using social media, or playing video games (VG) for entertainment for <2 h while sitting or lying down, over the past 7 days. Sleep duration was assessed through questions related to children's wake up and sleep times, while sleep quality was assessed based on a scale of 1 to 7. Parents reported the balance of their child's overall healthy movement behaviours compared with before COVID‐19 using a 5‐point Likert scale ranging from *a lot worse* (score = 1) to *a lot better* (score = 5).

Children were classified as meeting 24‐h movement guidelines (Bull et al., 2020; Okely et al., 2019; Tremblay et al., 2016) if they reported (per day): (1) ≥60 min of moderate‐ to vigorous‐intensity physical activity (MVPA); (2) ≤2 h of recreational ST; and (3) uninterrupted sleep for 9 to 11 h per night (Bull et al., 2020; Okely et al., 2019; Tremblay et al., 2016).

### 2.3 Statistical analyses

Data were downloaded from Qualtrics and manually checked and cleaned in Excel. Statistical analyses were carried out using SPSS software (version 27, Chicago, IL, USA). Sample characteristics were summarized using the mean and standard deviation and percentage of children meeting 24‐h movement guidelines. Pearson correlations were used to test associations between parent age and the time their child spent in PA, SB, using social media, and sleep. Differences between boys and girls in the time parents reported their child spent in PA, SB, using social media and sleep were assessed using independent samples *t*‐tests. Spearman's rank order correlations were used to assess associations between parent education level and income and time their child spent in PA, SB, using social media, and sleep. A 95% confidence interval for the percentages were calculated by using the formula of proportion:

$$p\pm z_{1-\frac{\alpha }{2}}\sqrt{\frac{p\left(1-p\right)}{n}}.$$

A forest plot was used to present parent‐reported changes in 24‐h movement behaviours of the children. Statistical significance was set at *p* < 0.05 for all analyses.

## 3 RESULTS

### 3.1 Parent and child characteristics

A total of 1021 parents completed the survey, of which 78.8% were Saudis. There were 2799 parents who started, but did not complete, the questionnaire. Parents were required to complete all questions before they could submit the survey. Fifty‐five per cent of respondents were mothers with an average age of 41 (±9.2) years. The mean age of the children was 8.5 (±1.85) years. Sixty per cent of the study sample were girls. Parents' average monthly income was $4355. One‐quarter of parents had a high school, 41% had a bachelor's degree, and 14% had a master's or PhD. Compared with the Saudi population, our sample of parents comprised more mothers and was slightly older. The monthly income and education levels were similar to the Saudi population (General Authority for Statistics, 2017, 2018, 2021) (Table 2).

**TABLE 2:** Characteristics of parents and children

Parents demographic profile		
Age (years), M (SD)		40.5 (7.58)
Parent/caregivers relationship to the child participating in the study, *n* (%)	Mother	565 (55.3)
	Father	456 (44.7)
Nationality, *n* (%)	Saudi	804 (78.8)
	Non‐Saudi	217 (21.2)
Education level, *n* (%)	Primary school	81 (8)
	High school	268 (26)
	Diploma	117 (11.5)
	Bachelor's degree	418 (41)
	Master's degree	101 (9.9)
	PhD	36 (3.6)
Living region, *n* (%)	Al‐Riyadh	477 (46)
	Al‐Jouf	35 (3.5)
	Al‐Qassim	63 (6.2)
	Al‐Bahah	10 (1)
	Asir	18 (1.8)
	Eastern Province	131 (12.9)
	Hail	28 (2.8)
	Jazan	8 (0.8)
	Mecca	79 (8)
	Medina	112 (11)
	Northern Borders	45 (4.5)
	Najran	6 (0.6)
	Tabuk	9 (0.9)
Monthly income (SAR), *n* (%)	0–3000	268 (26.3)
	3000–7000	186 (18.3)
	7000–10,000	112 (11)
	10,000‐15,000	237 (23.2)
	15,000‐20,000	155 (15)
	20,000+	63 (6.2)
**Child demographic profile**		
Age (years), M (SD)		8.5 (1.85)
Gender, *n* (%)	Boys	405 (39.7)
	Girls	616 (60.3)

### 3.2 Children's behaviours and changes in behaviours during COVID‐19

**FIGURE 1:**
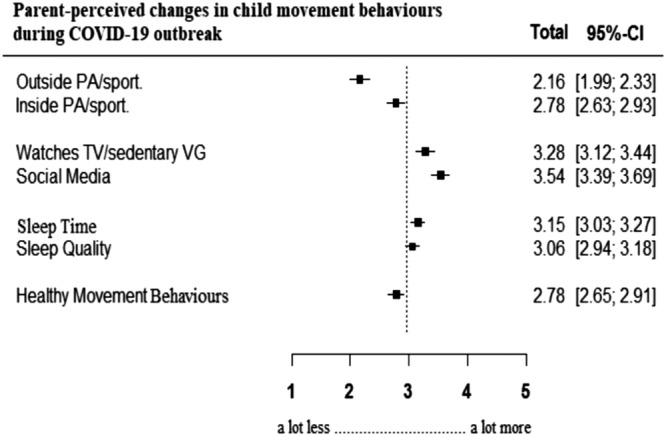
Forest plot of parent‐reported changes in 24‐h movement behaviours of Saudi children (6‐12 years) based on a 5‐point scale ranging from “a lot less” to “a lot more”. PA = physical activity. VG = video games

Table 3 shows the movement behaviours in Saudi children during the COVID‐19 outbreak compared with before COVID‐19. Only 3.4% (95% CI 0.00, 9.5) of children met the PA, SB, and sleep recommendations. Slightly more than half the children met the sleep recommendations (95% CI 52.9, 60.9), just over one third met the PA recommendations (95% CI 30.6, 40.8) and 15% met the SB recommendations (95% CI 9.5, 20.9). Boys' SB was significantly higher than girls (5.6 (2.15) versus 5.3 (2.20) hours, *p* = 0.013). For other behaviours, there were no significant differences between boys and girls. Sleep quality was reasonably high (average of 5.4 [2.26] points out of 7.0) and children played outside for 2.19 (1.34) h/day. Less boys than girls met the SB recommendations (13.1% [95% CI 4.0, 22.2] and 16.6% [95% CI 9.4, 23.8], respectively, *p* = 0.002). During COVID‐19, children spent less time in outdoor PA than before COVID‐19 (average of 2.16 [1.22] points out of 5.0) (Figure 1). Watching TV or playing sedentary VG and using social media were higher than before COVID‐19 (average of 3.28 [1.32] and 3.54 [1.48] points out of 5.0, respectively). Forty per cent of children had a screen device in their bedroom. Parents of girls perceived greater decreases in time spent in PA indoors (average of 2.85 points out of 5.0 [1.24]) (*p* = 0.037), greater increases in watching TV or playing sedentary VG (average of 3.49 points out of 5.0 [1.26]) (*p* < 0.0001), and greater increases in using social media (average of 4.08 points out of 5.0 [1.55]) (*p* < 0.0001) than boys.

**TABLE 3:** Children's movement behaviours during the COVID‐19 virus outbreak and compared with before COVID

	Total (*n* = 1021)	Girls (*n* = 616)	Boys (*n* = 405)	*p* value
**Children's movement behaviours, M (SD)**				
MVPA ≥60 min (days/week)	4.52 (2.40)	4.51 (2.39)	4.54 (2.41)	0.865
Activities to strengthen muscles and bones (number of days)	2.59 (2.37)	2.52 (2.34)	2.70 (2.42)	0.258
Sleep duration (h/day)	9.67 (2.26)	9.75 (2.42)	9.55 (1.99)	0.136
Sleep quality	5.40 (2.26)	5.38 (2.04)	5.42 (1.90)	0.697
Screen time ≤2 h/day (number of days/week)	5.39 (2.19)	5.26 (2.20)	5.60 (2.15)	**0.013**
Playing outside (h/day)	2.19 (1.34)	2.15 (1.32)	2.26 (1.35)	0.175
**Proportion of children meeting the WHO/Australian/Canadian guidelines (%) and 95% CI**				
MVPA	35.7 (30.6, 40.8)	32.6 (26.1, 39.1)	35.1 (27.2, 43.0)	**<0.0001**
Screen time	15.2 (9.5, 20.9)	16.6 (9.4, 23.8)	13.1 (4.0, 22.2)	**0.002**
Sleep^a^	56.9 (52.9, 60.9)	55.6 (50.3, 60.9)	58.8 (52.5, 65.1)	**<0.0001**
24 h combined	3.4 (0.00, 9.5)	3.8 (0.00, 11.6)	2.7 (0.00, 12.3)	**<0.0001**
**Perceived change in child movement behaviours during COVID‐19 outbreak, M (SD)**				
Physical activity or sport outside	2.16 (1.22)	2.19 (1.21)	2.11 (1.23)	0.293
Physical activity or sport inside	2.78 (1.24)	2.85 (1.24)	2.68 (1.23)	**0.037**
Watching TV or playing sedentary video games	3.28 (1.32)	3.49 (1.26)	2.96 (1.35)	**<0.0001**
Using social media	3.54 (1.48)	4.08 (1.55)	2.72 (0.87)	**<0.0001**
Sleep duration	3.15 (.94)	3.17 (.96)	3.12 (.90)	0.411
Sleep quality	3.06 (.94)	3.07 (.95)	3.04 (.92)	0.598
Overall healthy movement behaviours	2.78 (1.02)^b^	2.83 (1.01)	2.71 (1.04)	0.072

*Note*: 95% CI = confidence interval. Bold font indicates statistical significance.

Abbreviation: MVPA, moderate to vigorous physical activity.

^a^ Meeting sleep guideline (5–13 years).

^b^ Parents reported the balance of their children's overall healthy movement behaviours (sleep, SST and PA) as compared with before the COVID‐19 outbreak based on responses to a 5‐point scale ranging from *a lot worse* to *a lot better*.

Tables 4 and 5 show associations between parental demographic factors and children's movement behaviours. Parent age showed a significant positive, but weak correlation with the time their child spent using social media (*r* = 0.08, *p* = 0.007) (Table 4). No other associations between parent age and children's movement behaviours were significant.

**TABLE 4:** Pearson correlation analysis between parents age and children's movement behaviours

	Outside PA/sport	Inside PA/sport	Watches TV/sedentary VG	Social media	Sleep time	Sleep quality	Overall healthy movement behaviours
Parents age	−0.003	−0.01	−0.03	0.08**	0.02	−0.02	−0.01
	*p* = 0.93	*p* = 0.68	*p* = 0.25	*p* = 0.007	*p* = 0.38	*p* = 0.51	*p* = 0.81

^**^ Correlation is significant at the 0.01 level (2‐tailed).

**TABLE 5:** Spearman correlation between parent education level and income and child's movement behaviours

	Outside PA/sport	Inside PA/sport	Watches TV/sedentary VG	Social media	Sleep time	Sleep quality	Overall healthy movement Behaviours
Parents education	−0.06*	0.09**	0.17**	0.05	−0.01	−0.11**	−0.14**
	*p* = 0.04	*p* = 0.004	*p* < 0.0001	*p* = 0.09	*p* = 0.63		
Parents income	−0.06*	0.09**	0.15**	0.08**	0.029	−0.06**	−0.10**
	*p* = 0.04	*p* = 0.005	*p* < 0.0001	*p* = 0.009	*p* = 0.36	*p* = 0.049	*p* < 0.0001

*Note*: Parents education = primary school, high school, diploma, bachelor's degree, master's degree and PhD. Monthly income = 0–3000, 3000–7000, 7000–10,000, 10,000‐15,000, 15,000‐20,000, more than 20,000.

* Correlation is significant at the 0.05 level (2‐tailed).

^**^ Correlation is significant at the 0.01 level (2‐tailed).

The results of the Spearman correlations (Table 5) showed a significant, but weak negative correlation between parent education level and the time their child spent in outside PA/sport (*r* = −0.06, *p* = 0.04), and significant, but weak negative correlations in the quality of their child's sleep (*r* = −0.11, *p* < 0.0001) and in overall healthy movement behaviours (*r* = −0.14, *p* < 0.0001). Further, parent education had a significant, but weak positive correlation with the time their child spent in indoor PA (*r* = 0.09, *p* = 0.004) and in watching TV or sedentary VG (*r* = 0.17, *p* < 0.0001).

Parent income level (Table 5) showed a significant negative correlation with the time their child spent in outside PA/sport (*r* = −0.06, *p* = 0.04) and the quality of their child's sleep (*r* = −0.06, *p* = 0.049), and a significant, weak negative correlation in their child's overall healthy movement behaviours (*r* = −0.10, *p* < 0.0001). In addition, parent income level was positively correlated with the time their child spent in indoor PA (*r* = 0.09, *p* = 0.005) and in social media use (*r* = 0.08, *p* = 0.009), and a significant, weak positive correlation in watching TV or sedentary VG (*r* = 0.15, *p* < 0.0001). However, all of these correlations were weak.

### 3.3 New ways families are approaching movement behaviours

Almost half the parents (42%) indicated that their child was involved in more inside leisure activities during COVID‐19 than before COVID‐19. These included drawing (32.8%), playing VG (14.9%), playing soccer (13%), playing Intelligence Quotient games (11.1%), reading stories (6.1%). and yoga (4%). Few children were involved in sewing/crafts, house framing, and dancing (3%). Only 17% of parents indicated their child was involved in more outside leisure activity during the COVID‐19 outbreak. These included running (23.6%), hiking (17.2%), swimming (14.4%), visiting relatives (11.5%), martial arts (9.8%), photography (9.2%), biking (8.6%), and gymnastics (5.7%).

## 4 DISCUSSION

This is the first known study from an Arabian country to provide data on school‐aged children's 24‐h movement behaviour in relation to the COVID‐19 pandemic. The results indicate that children spent less time being physically active or playing outdoor sports, more time watching TV, or playing sedentary VG and more time sleeping. Girls were less active, engaged in more social media, and spent more time sleeping than boys.

Our findings are consistent with previous studies which have found that children's PA levels declined, and leisure screen time and social media use, as well as sleeping time increased during the COVID‐19 pandemic (Kovacs et al., 2021; Moore et al., 2020; Xiang et al., 2020). We found marked increases in time children spent in SB. It is likely these changes are a result of the government restrictions put in place due to COVID‐19, which limited children's opportunities to play outdoors (Kovacs et al., 2021). Moreover, as parents were working from home, they likely allowed their children to use electronic devices for longer periods of time to keep them busy as they worked (Okely et al., 2021). This finding may explain the significant increase in SB and the decrease in PA levels. School closures may be another reason for the decrease in children's PA levels. These may include not walking to school, which is associated with a decrease in PA levels (Faulkner et al., 2013).

Most children in the study did not have consistent bedtimes or wake‐up times during COVID‐19. As our data showed, on average children had later bedtimes (after 11 pm) than before COVID‐19. This may be explained by their increased use of screen devices in the 2 h before bedtime. However, the starting time for school was much later (15:30) during COVID when schooling was remotely delivered. This much later starting time likely explains the increase in sleep duration.

Moreover, 40% of children had a screen device in their bedroom, and this practice has been shown to be associated with lower sleep duration and quality (Chaput et al., 2014). Further, parents may be inadvertently influencing the quality of their child's sleep by using electronic devices during bedtime routines with their children (El Rafihi‐Ferreira et al., 2019; Fadzil, 2021). These sleep patterns are concerning as inconsistent wake‐up and bedtimes and inadequate sleep time may affect children's executive function (Warren et al., 2016) academic performance (Sun et al., 2019) and may have long‐term health consequences including hypertension, dyslipidaemia, weight gain, and metabolic syndrome (Medic et al., 2017). As children are influenced by the feelings and behaviours of their parents through observation and imitation (Bandura, 2008), modelling and encouraging these behaviours may help children meet the sleep and SB recommendations.

Internationally, a large proportion of children are not meeting the 24‐h movement guidelines (Roman‐Viñas et al., 2016). However, the percentage of Saudi children not meeting 24‐h movement behaviour guidelines was much lower than international studies conducted before the COVID‐19 pandemic. A multinational survey of school‐aged children to evaluate adherence to integrated movement behaviours in 12 countries showed that around 7% of children met the three recommendations (Roman‐Viñas et al., 2016).

The current study found that girls were less active than boys during the COVID‐19 pandemic. The decrease in PA levels can be partially explained by gender differences. For example, it has been reported that boys are more active than girls and that the gender difference in total amounts of activity is mainly due to gender differences in the amounts of self‐organized PA (Nielsen et al., 2011). However, it could also be due to lower levels of PE among girls in Saudi Arabia, as during COVID‐19 PE classes (remotely) were only provided in boys' schools.

Parents perceived a greater change in boys PA indoors compared with girls. This indicates that boys, in generally, were less active indoors than they were before COVID‐19. On the other hand, screen based indoor activities were more unfavourable among girls than boys. This could be explained by the distribution of girls' time during a 24‐h period. For example, the time spent in one behaviour could have displaced the time spent in another behaviour. In this study, girls slept more and spent less time in both MVPA and playing outside compared with boys; therefore, they had more time for indoor activities. Moreover, the culture and tradition of the Saudi society may encourage girls to spend more time watching TV and using social media as they have fewer opportunities to participate in PA compared with boys.

Parental age was positively, albeit weakly, associated with the time their child spent using social media. This could be explained by the differences in contemporary parenting compared with previous generations. Parents today may find it more challenging to control their children's use of social media. Moreover, as children are influenced by the feelings and behaviours of their parents (Bandura, 2008), the use of smartphones by parents could encourage children to use screen devices more (Hoyos Cillero & Jago, 2011; Ozturk Eyimaya & Yalçin Irmak, 2021).

Parents' education levels and income were weakly associated with children's movement behaviours change perceived by parents. In this study, children's sleep quality, the time spent in outdoor PA, and overall healthy movement behaviours decreased as parent education levels and income increased. Conversely, as parents' education level increased, the time their child spent in indoor PA and in watching TV or VG increased. A possible explanation of these results is that educated and high‐income parents could be more aware of the effects of COVID‐19 outbreak on their children's health. Therefore, they may have promoted their children's indoor PA level to reduce the effects of COVID‐19 outbreak.

As no Saudi 24‐h movement guidelines currently exist, we recommend the development of such guidelines for children in Saudi Arabia (Parrish et al., 2020). Furthermore, we suggest three strategies that may help Saudi children meet the 24‐h movement guidelines during the COVID‐19 outbreak. First, we recommend the Ministry of Education consider providing high‐quality online PE lessons. Kovacs et al. (2021) found that 57% of children who were active during online PE lessons also met the WHO PA recommendation. Also, when children revert to normal school days after the COVID‐19 pandemic, PE lessons should include a focus on time spent in PA during class for boys and girls to recover the decrease level of PA during COVID‐19 (Štveráková et al., 2021). This is especially important for girls, for who PE classes were only introduced in 2018, as a part of the Kingdom's vision (The Quality‐of‐Life Program). Second, parents should encourage their children to follow a structured daily schedule, which has been shown to be associated with higher odds of meeting ST and PA recommendations during the COVID‐19 (Kovacs et al., 2021). Finally, parents should encourage their children to participate in outdoor play to improve their PA levels (Kovacs et al., 2021; Tu et al., 2017). Parents should be encouraged to be more active with their children as they are a role model for their children (Brouwer et al., 2018).

To our knowledge, this is the first study in Saudi Arabia that investigated the cross‐sectional impact of COVID‐19 on movement behaviours among Saudi children (aged 6–12 years). A limitation of the study is that movement behaviours were subjectively captured via an online parent questionnaire; however, it was not possible to collect data using device‐based measures on a large sample due to the COVID‐19 pandemic. Using 24‐h movement guidelines from other countries is another potential limitation, highlighting the need to develop country‐specific guidelines for Saudi Arabia. Further, just over 70% of parents who started the questionnaire did not complete it, and it is not known if these parents differed from those who did complete the questionnaire.

## 5 CONCLUSIONS

The results of the current study demonstrate the impact of the COVID‐19 pandemic on Saudi children's 24‐h movement behaviours, prompting recommendations to parents, schools, and education decision‐makers to reduce the effect of the COVID‐19 outbreak and future pandemics on children's movement behaviours. Stakeholders are strongly encouraged to promote healthy levels of PA, SB, and sleep, by encouraging outdoor PA (where possible), minimizing children's use of screen devices when sedentary, educating parents about the importance of meeting movement guidelines by introducing national 24‐h guidelines, or promoting the WHO guidelines.

## CONFLICT OF INTEREST

The authors have no conflicts of interest to declare.

## AUTHOR CONTRIBUTIONS

Conceptualization, Y.A.A., A.‐M.P., and A.D.O.; methodology, Y.A.A., A.‐M.P., and A.D.O.; data collection and management, Y.A.A., formal analysis, Y.A.A., writing—original draft preparation, Y.A.A.; writing—review and editing, all authors. All authors have read and agreed to the published version of the manuscript.

## INFORMED CONSENT STATEMENT

Online informed consent was obtained from all subjects involved in the study.

## INSTITUTIONAL REVIEW BOARD STATEMENT

The study was conducted according to the guidelines of the Declaration of Helsinki. Approval was obtained from the Ministry of Education in Saudi Arabia (42640/2020) and the Human Research Ethics Committee at The University of Wollongong, Australia (HE288/2021).

## Supporting information




**Data S1.** Supporting Information

## Data Availability

The data that support the findings of this study are available from the corresponding author upon reasonable request.

## References

[cch12999-bib-0001] Al Hourani, H. , Alkhatib, B. , & Abdullah, M. (2021). Impact of COVID‐19 lockdown on body weight, eating habits, and physical activity of Jordanian children and adolescents. Disaster Medicine and Public Health Preparedness, 1–9. 10.1017/dmp.2021.48 PMC8129676 33588981

[cch12999-bib-0002] Bandura, A. (2008). Observational Learning. The international encyclopedia of communication.

[cch12999-bib-0003] Bates, L. C. , Zieff, G. , Stanford, K. , Moore, J. B. , Kerr, Z. Y. , Hanson, E. D. , Barone Gibbs, B. , Kline, C. E. , & Stoner, L. (2020). COVID‐19 impact on behaviors across the 24‐hour day in children and adolescents: Physical activity, sedentary behavior, and sleep. Children, 7(9), 138. 10.3390/children7090138 PMC7552759 32947805

[cch12999-bib-0004] Brouwer, S. I. , Küpers, L. K. , Kors, L. , Sijtsma, A. , Sauer, P. J. J. , Renders, C. M. , & Corpeleijn, E. (2018). Parental physical activity is associated with objectively measured physical activity in young children in a sex‐specific manner: The GECKO Drenthe cohort. BMC Public Health, 18(1), 1033. 10.1186/s12889-018-5883-x 30126399 PMC6102934

[cch12999-bib-0005] Bull, F. C. , al‐Ansari, S. S. , Biddle, S. , Borodulin, K. , Buman, M. P. , Cardon, G. , Carty, C. , Chaput, J. P. , Chastin, S. , Chou, R. , Dempsey, P. C. , DiPietro, L. , Ekelund, U. , Firth, J. , Friedenreich, C. M. , Garcia, L. , Gichu, M. , Jago, R. , Katzmarzyk, P. T. , … Willumsen, J. F. (2020). World Health Organization 2020 guidelines on physical activity and sedentary behaviour. British Journal of Sports Medicine, 54(24), 1451–1462. 10.1136/bjsports-2020-102955 33239350 PMC7719906

[cch12999-bib-0006] Chaput, J.‐P. , Leduc, G. , Boyer, C. , Bélanger, P. , LeBlanc, A. G. , Borghese, M. M. , & Tremblay, M. S. (2014). Electronic screens in children's bedrooms and adiposity, physical activity and sleep: Do the number and type of electronic devices matter? Canadian Journal of Public Health, 105(4), e273–e279. 10.17269/cjph.105.4511 25166130 PMC6972094

[cch12999-bib-0007] Child Labour in Arab States & International Programme on the Elimination of Child Labour (IPEC) (n.d.) Available online: https://www.ilo.org/ipec/Regionsandcountries/arab-states/lang--en/index.htm. (accessed on 8 January 2022).

[cch12999-bib-0008] El Rafihi‐Ferreira, R. , Pires, M. L. N. , & de Mattos Silvares, E. F. (2019). Behavioral intervention for sleep problems in childhood: A Brazilian randomized controlled trial. Psicologia: Reflexão e Crítica, 32(1), 5. 10.1186/s41155-019-0118-3 PMC6967177 32026011

[cch12999-bib-0009] Fadzil, A. (2021). Factors affecting the quality of sleep in children. Children, 8(2), 122. 10.3390/children8020122 33572155 PMC7915148

[cch12999-bib-0010] Farrag, N. S. , Cheskin, L. J. , & Farag, M. K. (2017). A systematic review of childhood obesity in the Middle East and North Africa (MENA) region: Prevalence and risk factors meta‐analysis. Adv. Pediatr. Res., 4, 8. 10.12715/apr.2017.4.8 29354689 PMC5773115

[cch12999-bib-0011] Faulkner, G. , Stone, M. , Buliung, R. , Wong, B. , & Mitra, R. (2013). School travel and children's physical activity: A cross‐sectional study examining the influence of distance. BMC Public Health, 13(1), 1166. 10.1186/1471-2458-13-1166 24330459 PMC3867216

[cch12999-bib-0012] General Authority for Statistics . (2017). Education and Training Survey 2017. https://www.stats.gov.sa/en/5656

[cch12999-bib-0013] General Authority for Statistics . (2018). Household Income and Expenditure Survey 2018. https://www.stats.gov.sa/en/37

[cch12999-bib-0014] General Authority for Statistics . (2021). Labor Force. https://www.stats.gov.sa/en/814

[cch12999-bib-0015] Guan, H. , Okely, A. D. , Aguilar‐Farias, N. , del Pozo Cruz, B. , Draper, C. E. , el Hamdouchi, A. , Florindo, A. A. , Jáuregui, A. , Katzmarzyk, P. T. , Kontsevaya, A. , Löf, M. , Park, W. , Reilly, J. J. , Sharma, D. , Tremblay, M. S. , & Veldman, S. L. C. (2020). Promoting healthy movement behaviours among children during the COVID‐19 pandemic. The Lancet Child & Adolescent Health, 4(6), 416–418. 10.1016/S2352-4642(20)30131-0 32458805 PMC7190292

[cch12999-bib-0016] Guerrero, M. D. , Vanderloo, L. M. , Rhodes, R. E. , Faulkner, G. , Moore, S. A. , & Tremblay, M. S. (2020). Canadian children's and youth's adherence to the 24‐h movement guidelines during the COVID‐19 pandemic: A decision tree analysis. Journal of Sport and Health Science, 9(4), 313–321. 10.1016/j.jshs.2020.06.005 32525098 PMC7276134

[cch12999-bib-0017] Han, E. , Tan, M. M. J. , Turk, E. , Sridhar, D. , Leung, G. M. , Shibuya, K. , Asgari, N. , Oh, J. , García‐Basteiro, A. L. , Hanefeld, J. , Cook, A. R. , Hsu, L. Y. , Teo, Y. Y. , Heymann, D. , Clark, H. , McKee, M. , & Legido‐Quigley, H. (2020). Lessons learnt from easing COVID‐19 restrictions: An analysis of countries and regions in Asia Pacific and Europe. The Lancet, 396(10261), 1525–1534. 10.1016/S0140-6736(20)32007-9 PMC7515628 32979936

[cch12999-bib-0018] Hoyos Cillero, I. , & Jago, R. (2011). Sociodemographic and home environment predictors of screen viewing among Spanish school children. Journal of Public Health, 33(3), 392–402. 10.1093/pubmed/fdq087 21047871 PMC3307230

[cch12999-bib-0019] Johns Hopkins University . (2021). COVID‐19 Map, Johns Hopkins Coronavirus Resource Center. https://coronavirus.jhu.edu/map.html

[cch12999-bib-0020] Kovacs, V. A. , Starc, G. , Brandes, M. , Kaj, M. , Blagus, R. , Leskošek, B. , Suesse, T. , Dinya, E. , Guinhouya, B. C. , Zito, V. , Rocha, P. M. , Gonzalez, B. P. , Kontsevaya, A. , Brzezinski, M. , Bidiugan, R. , Kiraly, A. , Csányi, T. , & Okely, A. D. (2021). Physical activity, screen time and the COVID‐19 school closures in Europe—An observational study in 10 countries. European Journal of Sport Science, 1–10. 10.1080/17461391.2021.1897166 33641633

[cch12999-bib-0021] Medic, G. , Wille, M. , & Hemels, M. E. (2017). Short‐ and long‐term health consequences of sleep disruption. Nature and Science of Sleep, 9, 151–161. 10.2147/NSS.S134864 PMC5449130 28579842

[cch12999-bib-0022] Mitra, R. , Moore, S. A. , Gillespie, M. , Faulkner, G. , Vanderloo, L. M. , Chulak‐Bozzer, T. , Rhodes, R. E. , Brussoni, M. , & Tremblay, M. S. (2020). Healthy movement behaviours in children and youth during the COVID‐19 pandemic: Exploring the role of the neighbourhood environment. Health & Place, 65, 102418. 10.1016/j.healthplace.2020.102418 32871499 PMC7455528

[cch12999-bib-0023] Moore, S. A. , Faulkner, G. , Rhodes, R. E. , Brussoni, M. , Chulak‐Bozzer, T. , Ferguson, L. J. , Mitra, R. , O'Reilly, N. , Spence, J. C. , Vanderloo, L. M. , & Tremblay, M. S. (2020). Impact of the COVID‐19 virus outbreak on movement and play behaviours of Canadian children and youth: A national survey. International Journal of Behavioral Nutrition and Physical Activity, 17(1), 85. 10.1186/s12966-020-00987-8 32631350 PMC7336091

[cch12999-bib-0024] Nielsen, G. , Pfister, G. , & Bo Andersen, L. (2011). Gender differences in the daily physical activities of Danish school children. European Physical Education Review, 17(1), 69–90. 10.1177/1356336X11402267

[cch12999-bib-0025] Okely, A. , Ghersi, D. , Loughran, S. , Cliff, D. , Shilton, T. & Jones, R. (2019). Australian 24‐hour movement guidelines for children (5‐12 years) and young people (13‐17 years): An integration of physical activity, sedentary behaviour. Retrieved from Canberra. https://www.health.gov.au/resources/publications/australian-24-hour-movement-guidelines-for-children-5-to-12-years-and-young-people-13-to-17-years-an-integration-of-physical-activity-sedentary-behaviour-and-sleep 10.1186/s12966-021-01236-2 PMC8734238 34991606

[cch12999-bib-0026] Okely, A. D. , Kariippanon, K. E. , Guan, H. , Taylor, E. K. , Suesse, T. , Cross, P. L. , Chong, K. H. , Suherman, A. , Turab, A. , Staiano, A. E. , Ha, A. S. , el Hamdouchi, A. , Baig, A. , Poh, B. K. , del Pozo‐Cruz, B. , Chan, C. H. S. , Nyström, C. D. , Koh, D. , Webster, E. K. , … Draper, C. E. (2021). Global effect of COVID‐19 pandemic on physical activity, sedentary behaviour and sleep among 3‐ to 5‐year‐old children: A longitudinal study of 14 countries. BMC Public Health, 21(1), 940. 10.1186/s12889-021-10852-3 34001086 PMC8128084

[cch12999-bib-0027] Ozturk Eyimaya, A. , & Yalçin Irmak, A. (2021). Relationship between parenting practices and children's screen time during the COVID‐19 pandemic in Turkey. Journal of Pediatric Nursing, 56, 24–29. 10.1016/j.pedn.2020.10.002 33181369 PMC7534794

[cch12999-bib-0028] Parrish, A.‐M. , Tremblay, M. S. , Carson, S. , Veldman, S. L. C. , Cliff, D. , Vella, S. , Chong, K. H. , Nacher, M. , del Pozo Cruz, B. , Ellis, Y. , Aubert, S. , Spaven, B. , Sameeha, M. J. , Zhang, Z. , & Okely, A. D. (2020). Comparing and assessing physical activity guidelines for children and adolescents: A systematic literature review and analysis. International Journal of Behavioral Nutrition and Physical Activity, 17(1), 16. 10.1186/s12966-020-0914-2 32041635 PMC7011603

[cch12999-bib-0029] Roman‐Viñas, B. , Chaput, J.‐P. , Katzmarzyk, P. T. , Fogelholm, M. , Lambert, E. V. , Maher, C. , Maia, J. , Olds, T. , Onywera, V. , Sarmiento, O. L. , Standage, M. , Tudor‐Locke, C. , Tremblay, M. S. , & ISCOLE Research Group . (2016). Proportion of children meeting recommendations for 24‐hour movement guidelines and associations with adiposity in a 12‐country study. International Journal of Behavioral Nutrition and Physical Activity, 13(1), 123. 10.1186/s12966-016-0449-8 27887654 PMC5123420

[cch12999-bib-0030] Sharara, E. , Akik, C. , Ghattas, H. , & Obermeyer, C. M. (2018). Physical inactivity, gender and culture in Arab countries: A systematic assessment of the literature. BMC Public Health, 18, 639. 10.1186/s12889-018-5472-z 29776343 PMC5960209

[cch12999-bib-0031] Simons, J. , Daly, D. , Theodorou, F. , Caron, C. , Simons, J. , & Andoniadou, E. (2008). Validity and reliability of the TGMD‐2 in 7–10‐year‐old Flemish children with intellectual disability. Adapted Physical Activity Quarterly, 25(1), 71–82. 10.1186/s12889-018-5472-z 18209245

[cch12999-bib-0032] Štveráková, T. , Jačisko, J. , Busch, A. , Šafářová, M. , Kolář, P. , & Kobesová, A. (2021). The impact of COVID‐19 on physical activity of Czech children. PLoS ONE, 16(7), e0254244. 10.1371/journal.pone.0254244 34237088 PMC8266068

[cch12999-bib-0033] Sun, W. , Ling, J. , Zhu, X. , Lee, T. M.‐C. , & Li, S. X. (2019). Associations of weekday‐to‐weekend sleep differences with academic performance and health‐related outcomes in school‐age children and youths. Sleep Medicine Reviews, 46, 27–53. 10.1016/j.smrv.2019.04.003 31060028

[cch12999-bib-0034] Tremblay, M. S. , Carson, V. , Chaput, J.‐P. , Connor Gorber, S. , Dinh, T. , Duggan, M. , Faulkner, G. , Gray, C. E. , Gruber, R. , Janson, K. , Janssen, I. , Katzmarzyk, P. T. , Kho, M. E. , Latimer‐Cheung, A. E. , LeBlanc, C. , Okely, A. D. , Olds, T. , Pate, R. R. , Phillips, A. , … Zehr, L. (2016). Canadian 24‐hour movement guidelines for children and youth: An integration of physical activity, sedentary behaviour, and sleep. Applied Physiology, Nutrition, and Metabolism, 41(6 (Suppl. 3)), S311–S327. 10.1139/apnm-2016-0151 27306437

[cch12999-bib-0035] Tu, A. W. , O'Connor, T. M. , Beauchamp, M. R. , Hughes, S. O. , Baranowski, T. , & Mâsse, L. C. (2017). What do US and Canadian parents do to encourage or discourage physical activity among their 5‐12 year old children? BMC Public Health, 17(1), 920. 10.1186/s12889-017-4918-z 29191203 PMC5710093

[cch12999-bib-0036] Valentini, N. C. (2012). Validity and reliability of the TGMD‐2 for Brazilian children. Journal of Motor Behavior, 44(4), 275–280. 22857518 10.1080/00222895.2012.700967

[cch12999-bib-0037] Warren, C. , Riggs, N. , & Pentz, M. A. (2016). Executive function mediates prospective relationships between sleep duration and sedentary behavior in children. Preventive Medicine, 91, 82–88. 10.1016/j.ypmed.2016.07.024 27477059 PMC5359977

[cch12999-bib-0038] World Health Organization . (2020). WHO Director‐General's opening remarks at the media briefing on COVID‐19. https://www.who.int/director-general/speeches/detail/who-director-general-s-opening-remarks-at-the-media-briefing-on-covid-19---11-march-2020

[cch12999-bib-0039] Xiang, M. , Zhang, Z. , & Kuwahara, K. (2020). Impact of COVID‐19 pandemic on children and adolescents' lifestyle behavior larger than expected. Progress in Cardiovascular Diseases, 63(4), 531–532. 10.1016/j.pcad.2020.04.013 32360513 PMC7190470

