# Supplementary material for: Impact of the COVID‐19 virus outbreak on 24‐h movement behaviours among children in Saudi Arabia: A cross‐sectional survey
Source: Child Care Health Dev. 2022 Mar 23:10.1111/cch.12999. Online ahead of print. doi: 10.1111/cch.12999 (PMC9111763; doi:10.1111/cch.12999)
Supplement: Supplementary file 1 — Data S1. Supporting Information [file CCH-9999-0-s001.docx]

**Supplementary material 1**

Associations between 24-hour movement behaviours (physical activity, sedentary behaviour, and sleep) and health among children in the Kingdom of Saudi Arabia

**PARENT QUESTIONNAIRE**

*This questionnaire is to be administered/completed by the MAIN caregiver of the child who lives with them and is 6 -12 years old at the time of interview. If you have more than one child aged between 6 - 12 years, please complete the questionnaire for the child whose birthday is next.*

| **MODULE OF CHILD AND CAREGIVER BACKGROUND** | | | | | | |
| --- | --- | --- | --- | --- | --- | --- |
| 1. **2.** | What is the date of birth of the child?  (dd/mm/yyyy)_________/__________/_______________ | | | | | |
| 1. **3.** | What is the sex of the child?  Boy  Girl | | | | | |
| **3.** | Parent/caregiver's relationship to the child participating in the study?  Mother  Father  Other (please specify): …………………………………………….. | | | | | |
| **4.** | In which region in Saudi Arabia do you live?  Riyadh  Makkah  Madinah  Qassim  Eastern región  Asir  Tabuk  Hail  The northern Border  Jazan  Najran  Al-Baha  Aljouf | | | | | |
| **5.** | What is the (parent/caregivers) date of birth?  (dd/mm/yyyy) _________/__________/__________________ | | | | | |
| **6.** | What is the highest level of parental education?  No formal schooling  Primary school  High school  Bachelor's degree  Master's degree  PHD degree  What is your monthly income (SAR)?  0-3000  3000-7000  7000-10,000  10,000-15,000  15,000-20,000  More than 20,000 | | | | | |
| **7.** | | What is your nationality?  Saudi  Non-Saudi | | | | |
| The next questions ask about your child's physical activity, sedentary behavior and sleep. | | | | | |  |
| **8.** | **Physical activity:**  Physical activity is any activity that increases your child’s heart rate and makes them get out of breath some of the time. Physical activity can be done in sports, school activities, playing with friends, walking to school, brisk walking, riding a bike, skateboarding or scootering safely, and helping to dig in the garden.  For the next question, please add up all the time your child spent in physical activity each day.   1. Over the past 7 days, on how many days was your child physically active for a total of at least 60 minutes per day?   Some examples of activities which strengthen muscles and bones are skipping, running, hopping and jumping, climbing or swinging on monkey bars, climbing frames, playing games like tug-o-war and hopscotch, doing structured activities like gymnastics and martial arts.   1. During the past 7 days, on how many days did your child do activities to strengthen their muscles and bones? 2. During the past 7 days, on average how much time per day did your child play outside? | | | | 1. days 2. day 3. days 4. days 5. days 6. days 7. days   7 days   1. days 2. day 3. days 4. days 5. days 6. days 7. days   7 days  0-< 1 hour  1-<2hour  2-<3 hours  3-<4hours  4-<5hours  6-<6hours  6 or more hours |  |
| **9.** | **Sedentary behaviour:**  Sitting or lying down, (with the exception of sleeping), are what we call ‘sedentary’ behaviours. You can be sedentary at work, at school, at home, when travelling or during leisure time. Sedentary behaviour requires little energy expenditure. Examples of sedentary behaviour include:  • Sitting or lying down while watching television or playing electronic games.  • Sitting while being a passenger in a vehicle, or while travelling on a bus or train.  • Sitting or lying down to read, study, write, or work at a desk or computer.  For the next question, please add up all the time your child spent watching TV/ videos/Internet using a smart phone or tablet or playing video or computer games for entertainment each day.  A - Over the past 7 days, on how many days did your child watch TV/ videos/ Internet using a smart phone or tablet or play video or computer games for entertainment for less than two hours while sitting or lying down? | | | | 1. days 2. day 3. days 4. days 5. days 6. days 7. days 8. days |  |
| **10.** | Does your child use electronic screen devices (e.g. TV, video game, computer, tablet or smartphone) in the 2 hours before bedtime on a daily basis?  Yes  No  Don’t know | | | |  |  |
| **11.** | **If Yes**, how close to bedtime does your child usually use these devices?  Closer than 30 minutes before bedtime  30 mins to less than 1 hour before bedtime  Between 1 and 2 hours before bedtime | | | |  |  |
| **12.** | Does your child have electronic screen devices in the room where he/she sleeps (e.g. TV, video game, computer, tablet or smartphone)?  Yes  No | | | |  |  |
| **13.** | How often do you use a smartphone to make calls, text messages, check email, watch a video during meals with your child?  Never  Less than once a week  Once a week  Most days  Every day  Don’t know | | | |  |  |
| **14.** | How often do you use a smartphone to make calls, text messages, check email, watch a video during bedtime routine with your child?  Never  Less than once a week  Once a week  Most days  Every day  Don’t know | | | |  |  |
| **15.** | **Sleep:**  What time did your child go to bed and turn the lights out to go to sleep last night?  What time did your child wake up today? | | | | ……pm  …….am |  |
| **16.** | On a scale of 1 to 7, with the higher number indicating higher quality, how would you rate the quality of your child's sleep?  1 would indicate very difficult to settle, wakes many times during the night for prolonged periods and is very restless (tosses and turns, throw off bedclothes) while 7 would indicate settles and drifts off to sleep with a few minutes, sleeps right through the night, and has a very sound, deep sleep)  1  2  3  4  5  6  7  Don’t know | | | | |  |
| **17.** | How many hours of sleep does your child get in a typical 24-hours day (including naps)? | | | …….hrs …….min | |  |
| **18a.** | Does your child have a consistent bedtime?  Yes, bedtime does not vary by more than 30 minutes each day  No, bedtime can vary more than 30 minutes each day | | **18b.** | Does your child have a consistent wake-up time?  Yes, wake-up time does not vary by more than 30 minutes each day  No, wake-up time can vary more than 30 minutes each day | |  |
| **19a.** | In the past three days, has your child: **Not** got enough sleep?  Yes  No | | | | |  |
| **19b.** | **If Yes**, was it because of: **(tick as many as appropriate)**  Outside noise (like traffic/train/street noises)  Indoor noise  Too Hot  Too cold  Too much light coming in to the room  Other (please specify): | | | | |  |
| **COVID-19 virus** | | | | | |  |
| **20.** | Has your child or a member of your household been diagnosed with COVID-19 during the past 6 weeks?  Yes  No | | | | |  |
| **21.** | **Compared to before the COVID-19 outbreak and related restrictions**, my child is doing physical activities or sport outside?  A lot less  A little less  About the same  A little more  A lot more | | | | |  |
| **22.** | **Compared to before the COVID-19 outbreak and related restrictions**, my child is doing physical activities or sport inside?  A lot less  A little less  About the same  A little more  A lot more | | | | |  |
| **23.** | **If your child is spending any time outside during the COVID-19 outbreak**, where are the common places this outside time is being spent?  Yard or driveway  Parks within walkable distance  Parks where you have to drive  Sidewalks, or neighbourhood streets  Other (Specify)  Not Applicable | | | | |  |
| **24.** | **Compared to before the COVID-19 outbreak and related restrictions**, my child watches TV, movies, uses the computer for leisure or plays sedentary video games?  A lot less  A little less  About the same  A little more  A lot more | | | | |  |
| **25.** | **Compared to before the COVID-19 outbreak and related restrictions**, my child uses social media?  A lot less  A little less  About the same  A little more  A lot more | | | | |  |
| **26.** | **Compared to before the COVID-19 outbreak and related restrictions**, my child sleeps?  A lot less  A little less  About the same  A little more  A lot more | | | | |  |
| **27.** | **Compared to before the COVID-19 outbreak and related restrictions**, my child’s sleep quality is?  A lot worse  A little worse  About the same  A little better  A lot better | | | | |  |
| **28.** | **Compared to before the COVID-19 outbreak and related restrictions**, the balance of my child’s overall healthy movement behaviours (i.e., physical activity, sedentary behaviours, and sleep) are?  A lot worse  A little worse  About the same  A little better  A lot better | | | | |  |
| **29.** | **As a result of the COVID-19 outbreak and related restrictions**, is there an inside leisure activity or hobby that your child is doing a lot more now?  Yes (please specify):  No | | | | |  |
| **30.** | **As a result of the COVID-19 outbreak and related restrictions,** is there an outside leisure activity or hobby that your child is doing a lot more now?  Yes (please specify):  No | | | | |  |
| **31.** | **As a result of the COVID-19 outbreak and related restrictions**, has there been a decrease in your child’s health (e.g., existing condition worsened or new condition developed)?  Yes (please specify):  No | | | | |  |

| **32.** | Comments:  ________________________________________________________________________________________________________________________________________________________________________  ____________________________________________________________________________________ |
| --- | --- |
| **33.** | Date survey was completed: DD/MM/YYYY ______/________/____________ |
